# Using Phenomenological Hermeneutics to Understand the Experiences of Managers Working with Quality Improvement Strategies in an Assisted Living Facility

**DOI:** 10.3390/healthcare7030087

**Published:** 2019-07-07

**Authors:** Jitendra Singh, Amy Wiese, Brandi Sillerud

**Affiliations:** School of Nursing & Healthcare Leadership, College of Science, Health & the Environment, Minnesota State University Moorhead, Moorhead, MN 56563, USA

**Keywords:** assisted living, Quality improvement (QI), managers

## Abstract

This qualitative research project aimed to gain an understanding of the experiences of managers who participated in the implementation of quality improvement projects in an assisted living facility. This study employed hermeneutic phenomenology as a research methodology, whereby managers working in an assisted living facility were invited to participate in a 60–75 min semi-structured interview. Six managers participated in the interviews. Five themes were developed from data analysis: (1) Quality improvement (QI) and resident-centered care go hand-in-hand; (2) Constant on-going commitment to continuous improvement is needed to implement QI; (3) Learning to communicate with team-members and residents/caregivers is important to implement QI; (4) Feedback is essential for the implementation of QI initiatives; and (5) Implementing new QI initiatives can be challenging. The managers emphasized the need for leadership commitment, the usage of standardized communication methods, and feedback strategies to ensure the success of QI initiatives. Additionally, the managers indicated that QI is directly related to resident-centered care and that efforts should be made to collect feedback from residents to further improve processes. Additionally, challenges surrounding the implementation of QI have been described. Since there is a scarcity of research on the implementation of QI methods in assisted living facilities, this study can provide practical tips to leaders and administrators.

## 1. Introduction

Assisted living facilities (ALF), one of the most important components of senior support services, provide a wide range of options to individuals who desire to live independently but need assistance with the activities of daily living. Research suggests that approximately one million Americans live in ALFs and this number will increase significantly by 2030 [1]. Advances in medicine, the adoption of evidence-based methods, and growth in technology has allowed individuals with chronic health problems to live longer, and further add to the demand for these services [2]. Approximately 75% of the residents living in ALFs need support with activities of daily living and 95% suffer from chronic health conditions. Further, nearly 33% of the residents in these facilities are hospitalized every year and 15% of the residents pass away or move to skilled nursing facilities to seek advanced care [3].

It is also important to note that there is a great need for this type of care since it is not provided by hospitals or clinics on a long-term basis. In 2014, there were 15,600 long-term care facilities with 1.4 million residents living in them [4]. It is important to make sure this type of care is available in order to ensure that people who are unable to perform daily functions are receiving the care that they need and deserve.

Evidence suggests that there is a rise in the demand for assisted living facilities and the majority of the U.S. population will need this type of care during their lifetime. However, there is an increasing recognition that these facilities struggle to provide high-quality services to residents [5]. The application of quality improvement strategies may enhance the quality of the care provided to the residents of these facilities [6]. Managers play a major role in the implementation of quality improvement methods in health care facilities, including assisted living facilities [7]. As quality problems and errors in the process of care continue to grow, it is extremely important to gain an understanding of the experiences of managers who have participated in the implementation of quality improvement projects in these facilities. This may help identify critical success factors and challenges surrounding quality improvement (QI) implementation in these facilities.

### 1.1. Background and Rationale

QI can be defined as a collaborative effort led by administrative professionals, clinical workers, patients, caregivers, and researchers to improve processes and health outcomes for patients/residents who need care. Key aims, described by the Institute of Medicine (IOM), such as patient safety, efficiency, patient-centric, timeliness of services, and effectiveness of care should also be considered as managers plan to implement QI strategies in health organizations [8,9]. 

Evidence suggests that managers need to make efforts to clarify and understand problems, involve key individuals in process change initiatives and continuously monitor progress in order to successfully implement QI initiatives [10]. Improving the standard of quality enhances the lives of residents in long-term care facilities. Quality improvement methods allow long-term care facilities to achieve increases in quality, safety, and efficiency, which all enhance the lives of the residents living in these facilities [11]. Managers are the connection between upper administration and frontline workers in an organization, which makes them responsible for many activities to fulfill their positions [12]. One of their roles is to motivate staff in the implementation of quality improvement initiatives [13]. Middle managers not only play a major role in managing day-to-day activities, but also provide primary support to frontline staff [7]. A manager’s role of motivating and supporting frontline staff is an essential element to a successful quality-improvement process since frontline staff interact with residents daily at long-term care facilities.

Managers are also involved in creating a culture of change, which supports QI in long-term care facilities. It is important to note that managers need to take several steps to create real-time participation and staff involvement in the process of QI. Efforts should be made to share hard data and patient stories to enhance the emotional involvement of employees in the QI initiatives. Evidence-based initiatives with a clear plan on how new changes will be implemented are needed to create buy in from the employees. Further, managers should use open channels of communication to ensure that employees understand the connection between the new initiative and the intended outcome. It may be difficult for staff to understand the full QI initiative as they start working on a new project, therefore, managers should work with leaders to identify appropriate training needs and other requirements (time, education programs) for employees [14].

Managers can use their unique position between upper administration and frontline workers in an organization to create lasting change [12]. This unique position allows them to motivate frontline staff to support this change. Middle managers support change implementation by performing four tasks: distributing adequate information, gathering information, mediating between strategy and regular activities/tasks in the organization, and creating an environment where employees actively participate in innovative change initiatives [15]. These four tasks, along with their unique position in a facility, help create change in the facility and support a new culture that utilizes quality improvement to improve resident outcomes. 

Prior research suggests that managers can play a significant role in enhancing quality and safety initiatives, which in turn can lead to improved care for patients [16]. Although several studies have focused on the role of managers in the quality improvement process, the emphasis has been largely on acute care facilities and hospital settings [16,17,18]. While it is important to focus on managers who work in acute care and hospital settings, efforts should also be made to gain an understanding of the experiences of managers who work with quality improvement projects (or with quality and safety initiatives) in ALFs. Dissemination of the findings from this research could help management and senior leaders (in a senior care environment) as they work on implementing new QI programs in their organizations.

### 1.2. Purpose Statement

This qualitative research project aimed to gain an understanding of the experiences of managers who participated in the implementation of quality improvement projects in an assisted living facility. More specifically, the objectives were to: (a) understand how managers define the quality of patient care and administrative processes; (b) understand the importance of leadership support and education needed to implement QI strategies; and (c) explore the challenges surrounding the implementation of QI strategies in assisted living facilities. 

## 2. Materials and Methods 

### 2.1. Research Methodology 

Hermeneutic phenomenology was selected as a suitable research methodology for the current study. This methodology allows interpretation and analysis of textual information in order to enhance the understanding of the meaning of day-to-day experiences of research participants [19]. Hermeneutic research methodology is “aimed at producing rich textual descriptions of the experiencing of selected phenomena in the lifeworld of individuals that are able to connect with the experience of all of us collectively” [20,21]. Because phenomenology deals with the experience of individuals, this approach was used for understanding the experiences of managers who were involved in the implementation of quality improvement projects in an assisted living facility. The approach was hermeneutic because it allowed researchers to explore and interpret these experiences in light of what they already knew about the topic under consideration (practical and theoretical knowledge) [21,22]. The unique feature of the study was that one of the researchers in the project had significant experience in the implementation of quality improvement projects in skilled nursing facilities. This researcher was trained in Lean quality improvement methodology and played a key role in the implementation of Lean methodology at several skilled nursing facilities in the U.S. The implementation of Lean methodology at these skilled nursing facilities resulted in an improvement in the quality, safety, and efficiency of resident services and administrative processes. The other researcher had experience in clinical and process improvement initiatives in an acute care hospital. These real-world experiences allowed researchers to build trust and communicate in the same jargon as the one used in the facility. The researchers also stepped back to think about the meaning of the state of affairs and collected data rather than “accepting pre-conceptions and interpretations at the face value” [21] (pp.620).

The researchers engaged two research assistants in this project. One student (research assistant) was charged with the recruitment of participants, the distribution of consent forms, scheduling interviews, and helping with data collection for the study [22]. The second research assistant completed an in-depth study of qualitative research methods and assisted with data analysis of the project. Approval to conduct the study was obtained from the Office of Research Ethics at the university. The use of pseudonyms, the careful selection of examples, and the appropriate modification of participant-specific information helped ensure that participant privacy was protected [23].

### 2.2. Research Participants

A homogeneous purposive sampling strategy was used to recruit participants, as this allowed researchers to select individuals who had past experience in implementing and/or working with QI projects in the assisted living facility. Individuals who had more than five years of experience in the implementation of QI strategies were selected to participate in this study. This strategy helped in achieving a homogeneous sample of participants with experience in QI projects [24]. Six managers with an average of 13 years of experience in QI projects were selected to participate in the study. On average, the managers had 18.8 years of experience in ALFs. These individuals were recruited with the support of organizational leaders (for participant characteristic, see Table 1). It is important to note that these individuals worked in positions that involved managerial tasks and responsibilities. They were charged with achieving goals that needed planning, organizing and dealing with staff members to achieve desired organizational objectives. They also had a significant involvement in the implementation of QI projects and provided support to the leadership of the organization [25,26]. All the individuals who agreed to participate in the project completed the interviews.

### 2.3. Data Collection and Data Analysis

This research aimed to understand the experiences of managers who participated in the implementation of QI projects in an assisted living facility and to produce a detailed account of the significance (and overall meaning) of those experiences [27]. One research assistant and researchers participated in bracketing thoughts prior to the beginning of the data collection. Bracketing allows researchers to put aside prior knowledge, thoughts, and experiences in order to describe collected data [28]. Because one of the researchers had a significant background in the application of QI strategies in a long-term care organization, bracketing allowed the researcher to put aside pre-existing ideas/thoughts, attitudes and/or opinions about the topic (e.g., how QI strategies should be implemented and problems faced when QI strategies are implemented in long-term care settings). Bracketing helped to minimize the influence of potential bias [22,28]. Two email invitations were sent to the eligible participants. These invitations also included information about the study’s objectives, benefits/risks, and the overall time needed to complete the interview. Once responses were received from participants, a convenient/preferred location was chosen in consultation with the participants. Written consent from participants was also obtained prior to the interview meetings. Each participant completed a semi-structured interview that lasted 60–75 min. The interviews were conducted by the researcher who had knowledge about long-term care facilities. The interviews were audio-taped and transcribed verbatim. Written transcripts were sent to all the participants for review to ensure that the information was adequately represented in the written format. The interview guide was written once a literature review related to quality improvement initiatives in senior care facilities was completed. Researchers also met several times to discuss the guide and its overall relevance to the research questions. The interview guide included questions such as: tell us what quality improvement means to you; the importance you place on QI in your organization; leadership support needed to facilitate the implementation of QI projects; and challenges encountered while implementing QI projects. Field notes were also taken during the data collection phase and were entered as data for the study. These notes include a description of the interview environment and thoughts of the researchers who conducted the interview. This allowed researchers to return to the interview setting (in concept rather than actually visiting the site) while conducting an analysis of the collected data [19,29].

Once the interviews were completed, the entire data was transcribed verbatim by the researcher with experience in a skilled nursing facility and the second research assistant. Both individuals also heard and reviewed the recorded data several times as they worked on data analysis for the study. This allowed researchers to be immersed in the data and think about what participants actually said/meant during the interviews. Following this step, researchers reached out to participants for additional clarification. Final transcripts were also sent to participants to ensure that data was captured accurately. After approval was received from participants, the researchers highlighted the statements that appeared to reveal the topic under consideration. An inductive approach to thematic analysis was utilized in the current project. The researchers reviewed each line of the transcripts and the field notes several times. Labels were used to highlight key phrases (e.g., resident-centered care, process improvement). As the process continued, phrases were compared to determine whether they fit under the already existing code or whether new codes needed to be developed. The phrases that represented the same concept were gathered into key thematic statements. Quotes from participants were used to support these themes [19]. After an initial round of analysis, the researcher met with the other members of the team who had expertise in qualitative research methods to discuss themes and to identify areas that needed more inquiry. Following the meeting, themes were further refined and all the interviews/transcripts were revisited to examine key commonalities and differences. This led to the identification of final themes that described the experiences of participants with quality improvement initiatives in the assisted living facility [30,31]. The final analysis was confirmed by the research team.

### 2.4. Rigor 

Several measures were adopted to enhance the trustworthiness of the study/research procedures. In order to ensure credibility, the researchers took time to establish a rapport with the participants prior to beginning the study. Adequate time was allocated for each interview and the location of each interview was chosen in collaboration with participants so that they could share their thoughts freely. In addition, the researchers took notes during the interview sessions, collected appropriate demographic information, and audio-taped each interview. Each interview was transcribed and the transcripts were sent to the participants to ensure that data was accurate. These procedures helped to enhance the reliability of the findings of the study. Furthermore, the use of homogenous purposive sampling and the inclusion of necessary details with rich textual descriptions helped to increase the trustworthiness of the overall process [32,33]. 

## 3. Results

Six managers with extensive experience in quality improvement projects participated in the study. Five themes emerged from the data analysis: (1) Quality improvement and resident-centered care go hand in hand; (2) Constant on-going commitment to continuous improvement is needed to implement QI; (3) Learning to communicate with team-members and residents/caregivers is important to implement QI; (4) Feedback is essential for the implementation of QI initiatives; and (5) Implementing new QI initiatives can be challenging.

### 3.1. Theme 1: Quality Improvement and Resident-Centered Care Go Hand in Hand

The managers working with QI projects reported that several mechanisms have been put in place to provide high-quality and safe care to residents. Attention to the needs of residents, the ability to offer different care options at reasonable prices, and treating residents with respect were recurring themes in almost all the interviews. For example, Deborah indicated:

“The world of retirement communities is ever evolving and as times goes on, we are going to see the interest change of people that we serve. They are going to be looking for different events and activities, different services, different inclusions, various price points. So working to overall to offer a good product to meet the needs of our people at the time and being welling to change that.”

The managers also indicated that it is important to improve the quality of care and focus on a resident-centered approach, as it allows the facility to meet its financial goals. Supporting this claim, Peggy indicated:

“Quality to me is the reflection of how happy our residents are, and how happy our caregivers are. If both are happy and satisfied, we are going to have a full building and we are going to meet our financial goals.”

The managers who were not directly involved with resident care also understood the importance of resident-centered care in the facility. They reiterated the importance of dignity, home-like treatment and integration of the best practices to provide high-quality services to residents. This is illustrated by Barbara’s statement:

“As far as resident care, that’s always a big thing I’m not personally directly involved in resident care but working with our staff who are, our nurses, and CNAs. In resident care too it is the same honor, and respect of their dignity. We don’t want just to enter an apartment without knocking or without asking, or without saying who we are. It is their home, and we want to treat it like their home, and like as if it our home, and someone is coming to us. We have looked at several quality improvement tools and consistently try to implement methods that fits with our facility.”

Supporting this claim, Sharon, who had 15 years of experience in quality improvement, indicated:

“One of the things that is part of our efforts to improve customer satisfaction and implement hospitality is this whole list of standards that is kind of similar to the Ritz Carlton has done in the hospitality industry. Creating this list of standards has really provided a guide for quality in many ways. I think what I see with this program and future efforts is more of a focus on quality and providing consistent guidelines for quality for all of our communities, where maybe that didn’t exist in the past.”

In addition to resident care processes in the facility, the managers were also attentive to move-in processes and wanted to promote smooth transitions when new residents moved into the facility. For example, Peggy indicated:

“We want that experience to be the best possible for patients or for residents when they move in. We want it to be smooth and seamless when they move in, so if we have our nursing team involved, we are going to be communicating with them as much as possible, so they know as much information about the new resident so that they can have a successful transition to our community. We have looked at several process improvement tools to facilitate successful transition of residents to our facility.”

### 3.2. Theme 2: Constant On-Going Commitment to Continuous Improvement Is Needed to Implement QI

The managers indicated that process improvement and quality improvement initiatives take time and that efforts should be made to show that leaders (such as chief executive officers) are supportive of these change initiatives. Leaders should demonstrate a clarity of vision and must be able to communicate effectively across the organization. Furthermore, the managers also indicated a need for setting goals and allocating resources to ensure that employees have full support from leadership.

This is illustrated by comments made by Barbara. When asked about support, she indicated:

“Process improvement and quality improvement take time and so making sure we find the time and that we are making the right steps to get there too. Setting goals is a big thing, if we set a goal to have this done by X date that helps, making sure that we keep that in mind to know how we can divide the work out so it is not totally overwhelming. Those are probably the two biggest things, time and money.”

Corroborating this claim, Sharon indicated:

“I can’t say enough good things about that support, they are very supportive, very trusting in me, they let us spread our wings so there is a lot of support first hand that way as far as going in process improvement. They are definitely a big supporter. I would say as far as the support of our organization on QI deals with how you communicate. I do believe that our executive director is very open and supportive person just naturally, but there is also a way you need to her in order to do these projects. The projects do cost money. The objects that we developed for the project were $10,000 in piece. There is a lot of time and a substantial amount that goes into these projects so there is that times of (…). She is under the understanding that if something fails we did not lose the game but we are better because of it.”

When asked about training, resources, and recognition of employees as they work on implementing QI strategies in processes, the managers indicated that the organization provides a lot of support, additional training, and recognition to employees who participate in quality improvement projects and major change initiatives. The managers indicated that the organization utilized lists of guidelines/standards from the hospitality industry that served as a guide for quality in many ways (see Sharon’s comment in Theme 1).

### 3.3. Theme 3: Learning to Communicate with Team-Members and Residents/Caregivers Is Important to Implement QI 

The managers felt that communication between team members (staff members) is extremely important to implement QI initiatives. Appropriate, timely, and consistent communication was found to be effective when dealing with or implementing QI strategies. The managers indicated that constant communication between different departments was helpful to learning what others were doing to improve day-to-day processes. A need for a standardized communication format was also highlighted during the interviews. All but one or two managers mentioned that a standardized format of communication was needed to support and foster the process of QI. 

For example, Shawn indicated:

“I think communication is huge. It really is the key to effective organization, and making sure that everybody needs to know something is in the know at the right time. I don’t think you can over communicate that is my philosophy on it; I would rather more than I need to than not enough to get my job done. Communication in my opinion is essential.”

The need for a standardized communication format was also highlighted during the interviews. Almost all the managers mentioned that a standardized format of communication was needed to support and foster the process of QI. Peggy mentioned:

“I think we need a method or plan that all the employees should follow. This will improve communication across departments and also with community. I do not want to confuse my employees with inconsistent messages of format does not make sense. We have tried to develop a template for communication in this organization.”

It is important to note that the managers highlighted the importance of communication between team-members and residents/caregivers. Including residents and/or caregivers in major initiatives and using their feedback to make changes was considered extremely important. The managers also indicated that care planning was completed in consultation with resident/caregivers and efforts were made to communicate clearly in case there were changes.

### 3.4. Theme 4: Feedback Is Essential for the Implementation of QI Initiatives

Nearly every manager highlighted the importance of feedback and data collection to ensure QI initiatives had been implemented appropriately. The managers also highlighted the importance of making changes/adjustments based on the feedback they receive from employees and residents. Barbara indicated:

“Quality improvement is not a race; there is no finish line. It is extremely important to collect data to see how you are going. Data collection should be a never ending process. Quite frankly, without data and feedback, a lot of people here have no idea how to improve. I think it’s always a challenge to continue to improve with QI so it is hard work but it definitely get you to where you want to be at the end game.”

The managers also indicated that the organization utilizes a morning standup meeting for gathering feedback. All the employees were encouraged to attend these meetings and provide feedback on new initiatives and/or existing processes in the organization. In addition to this, each department organized standup meetings for their staff members. All the staff members (in the respective department) were required to attend these standup meetings. The importance of a resident council and resident meetings was also emphasized. The managers mentioned that leadership used these meetings to collect feedback from residents and care givers.

For example, Jennifer said:

“We have resident council once per month and then followed by a resident meeting. A lot of the same topics could be shared in the residents meeting. I think our executive director always keep a list of items or topics to talk about each meeting each month. We do have a suggestions box that the resident can anonymously or identified leave a comment or suggestion.”

These claims suggest that data collection is not only important from a QI standpoint, but also helps to ensure that the needs of the residents are heard. The managers also emphasized the importance of resident satisfaction surveys and indicated that these scores were considered as the highest indicator of whether the organization succeeded in providing high-quality services to customers. 

For example, Shawn said:

“I would say we take our resident satisfaction survey scores very seriously. To me, that’s the biggest indicator of whether we are successful or not. We really encourage every resident and family members to participate, we actually have a large meeting that we invite everybody to come, but those who haven’t filled it out, we actually go door-to-door and try to get their feedback. We would love if they give us great scores, but we want honest feedback, and we would like the comments that they provide too because that really gives us a road map of what our goals should be for the coming year.”

### 3.5. Theme 5: Implementing New QI Initiatives Can Be Challenging

The managers working in the assisted living facility reported that new change initiatives can lead to frustration, unnecessary anxiety, and resistance, unless people see the benefits of these initiatives. A key finding that was present among all the managers was the fear of the unknown and resistance to change. This fear originated from when the organization tried to implement new QI initiatives in resident-care administrative processes. For example, Peggy indicated:

“*On occasion you have people who really don’t want to change the way they’ve always done something, even though you can prove that it’s going to make their life easier, and our results will be better. Sometimes if people just feel like they have always done something and it worked, they don’t want to change. I think really trying to encourage those people to think outside the box other, we want their feedback but we also need to get them on board for change*.”

Peggy continued:

“*We completely change the way that we have always done things and for employees who has been doing that for 8,9,10 years, we completely kind of shock their world. Thankfully, when we started getting through the most painful parts, we started to see them come on board. It was challenging*.”

Supporting this claim, Shawn indicated:

“*There was a lot of anxiety in our team and I think a lot of negativity that we work through it after the project started, I think it was just the fear of the unknown*.”

## 4. Discussion

This research aimed to understand the experiences of managers who participated in the implementation of quality improvement projects in an assisted living facility. We found that managers in assisted living facilities placed a high importance on quality, resident-centered care processes.

A reoccurring theme in this research is that quality and resident-centered care go hand-in-hand. Happy residents equate to positive financial outcomes. If organizations have happy residents, they have full organizations, which creates positive financial outcomes. With healthy margins, an organization can invest in operational and capital expenditures and grow. Managers and organizations can take lessons from other industries, such as hotels, to improve hospitality and provide an exceptional experience, similar to a hotel stay [34]. Resident-centered care starts with respect and trust. Considering the nature of this environment, the residents’ homes need to be respected. This includes knocking and building trust when entering the residents’ homes. Standardization of processes, such as the move-in process, needs to be seamless. This is an area that quality improvement can impact through standardized processes to ensure an easy transition into the assisted living facility. These findings can be related to a recent review in which authors reported that attention to the needs of residents and close relationships between the caregiving team and the residents is crucial for enhancing the quality of the care provided to residents in nursing facilities [35,36]. 

This study demonstrates that organizations must have a constant, on-going commitment to continuous improvement in order to implement QI. Change is ever present in healthcare. It is imperative that managers utilize standardized processes and quality improvement methods to help set goals and allocate resources to support staff through change. These findings align with the results of a recent study that demonstrated that organizations must provide a sufficient structure and enhance opportunities for collaboration and teamwork to sustain the culture of continuous improvement [37]. 

The findings indicate that learning to communicate with team members and residents/caregivers is important during continuous-improvement activities. Like many change processes, communication is essential for ongoing and continuous quality improvement. This communication needs to be between managers and staff and between different departments within the organization. Interdepartmental communication ensures the prevention of the occurrence of an information silo and provides the ability to learn from other staff within the organization. If we fail to listen to others within our organization or outside of our own departments, we may be limited in our innovations. Sometimes, we may find the answer to a problem within a different department in our own organization; we just need to communicate outside our department. This was illustrated in a study where researchers demonstrated that open communication between providers and patients and across different departments is needed for safe and effective patient care processes [38]. 

The research findings suggest that there is a need for standardized communication formats to ensure that departments can learn from each other to improve day-to-day processes. Staff prefer frequent communication at appropriate times for support in their jobs and QI processes. Managers need to encourage staff input during all QI processes to ensure the successful completion of the goals associated with each new and ongoing process. Staff can contribute new ideas and innovations that managers may not have thought of if communication is on-going and encouraged. Managers should develop tools to support this communication, such as templates. These templates can help standardize processes and ensure that communication is consistent. Templates for communication during QI processes could parallel the nursing care plan for residents. These care plans are collaborative approaches to care. Templates for communication during continuous quality improvement processes should ensure collaborative decision-making between managers and staff. This will help ensure that all members of the team are in agreement and working toward a common goal. This was also found in a study where researchers indicated that it is vital to follow a standard communication plan and involve staff in shared decision-making, as this helps to improve the quality of care for residents [10]. An effective clinical and administrative communication plan is required for the management of health care services. Accurate, timely, clear, and honest communication and information sharing between providers lead to high-quality patient-centered care [39].

Feedback is essential for the implementation of QI initiatives. Data needs to drive decision-making and QI processes within organizations. Data needs to be collected prior to any QI process, during the process, and after implementation. This data needs to be analyzed, with appropriate feedback given to staff, and included in the QI process. Using tools like Plan, Do, Study, Act (PDSA) supports this concept of the utilization of data with feedback to drive QI processes. Using the PDSA allows managers to evaluate the data and processes and provide feedback to either maintain, adjust, or abandon the process based on the data. It is important to collect data on a regular basis, as this allows us to understand whether we are headed in the right direction [40]. 

Feedback can also come from staff and residents. Staff need to provide feedback on QI processes. Methods of providing this feedback can include huddles, stand-up meetings, department meetings, and one-on-one communication with managers. Residents can provide feedback through organized resident council meetings and one-on-one communication with staff. Another method for collecting feedback is through resident satisfaction surveys. These surveys can provide important data regarding processes and resident satisfaction within an ALF. It is important to encourage resident and family participation in those surveys to gather adequate data upon which to base decisions. If organizations take the time to collect data and feedback, then they must respond to positive and negative feedback obtained via the surveys. Feedback from residents and staff members not only serves as an additional source of data, but also helps in planning new QI initiatives. Recent literature demonstrated that patient and staff feedback is essential for planning and for the success of QI initiatives. Efforts should be made to include the feedback and work on “co-designing” the process in partnership with patients and staff members [41]. 

Implementing new QI and change initiatives can be challenging. The fear of change is a common phenomenon in every organization. This is also true for health care organizations, including ALFs. Managers have a significant role in quality improvement initiatives. While several studies have been conducted in hospitals and acute care organizations, there lacks research that aims to understand the experiences of managers in QI initiatives in assisted living facilities. This study demonstrates that managers in assisted living facilities place a high importance on QI initiatives. The importance of on-going communication between providers, feedback from employees and residents, and on-going commitment to continuous improvement is also highlighted in the current study. This study suggests that managers, staff, and residents should work toward the same vision for continuous quality improvement within an organization.

## 5. Limitations 

There were several limitations in this study. First, the researchers deliberately focused on managers who had several years of experience in implementing QI projects in the facility. Second, managers were recruited from one facility due to time and budget constraints. Third, only six managers participated in the current study. Recommendations for future studies would be to attempt to recruit additional participants (experienced and novice managers) and include similar facilities, as this will help to gain further insights into the experiences of managers in QI projects. 

## 6. Conclusions

This project allowed the researchers to begin understanding the experiences of managers in assisted living facilities and to gain insights into how they define quality, the importance of leadership, support, and education needed to implement QI methods, and to explore challenges while implementing these methods. The managers indicated that QI and resident-centered care go hand-in-hand, leadership support is needed for continuous improvement initiatives, and feedback is essential for the implementation of QI initiatives. Further, the importance of communication in QI was described. These findings can help leaders and administrators as they plan implementations of QI initiatives in their organizations. 

## Figures and Tables

**Table 1:** Description of participants.

Participants (Pseudonym)	Gender	Age (Years)	Experience with QI (in Years)	Experience in ALF (in Years)	Role	Area of Work	Major QI Projects
Peggy	Female	52	10	14	Coordinator	Resident Services	QI in coordination of services for residents
Deborah	Female	48	12	20	Administration	Administration of the facility	Overall QI in the facility
Barbara	Female	57	16	28	Marketing	Marketing	QI in marketing
Shawn	Male	45	12	15	Manager	Resident Care	QI in resident care
Sharon	Female	46	15	17	Assistant Manager	Admissions and Discharge	QI in resident care
Jennifer	Female	51	14	19	Manager	Clinical care	QI in resident care
